# Dysfunction of attention switching networks in amyotrophic lateral sclerosis

**DOI:** 10.1016/j.nicl.2019.101707

**Published:** 2019-02-02

**Authors:** Roisin McMackin, Stefan Dukic, Michael Broderick, Parameswaran M. Iyer, Marta Pinto-Grau, Kieran Mohr, Rangariroyashe Chipika, Amina Coffey, Teresa Buxo, Christina Schuster, Brighid Gavin, Mark Heverin, Peter Bede, Niall Pender, Edmund C. Lalor, Muthuraman Muthuraman, Orla Hardiman, Bahman Nasseroleslami

**Affiliations:** a Academic Unit of Neurology, Trinity College Dublin, The University of Dublin, Ireland; b Trinity Centre for Bioengineering, Trinity College Dublin, The University of Dublin, Ireland; c Beaumont Hospital Dublin, Department of Neurology, Dublin, Ireland; d Beaumont Hospital Dublin, Department of Psychology, Dublin, Ireland; e Computational Neuroimaging Group, Trinity College Dublin, The University of Dublin, Ireland.; f Trinity College Institute of Neuroscience, Trinity College Dublin, The University of Dublin, Ireland.; g Department of Biomedical Engineering, University of Rochester, Rochester, New York, USA.; h Movement Disorders and Neurostimulation, Biomedical Statistics and Multimodal Signal Processing Unit, Department of Neurology, Johannes-Gutenberg-University Hospital, Mainz, Germany

**Keywords:** Amyotrophic lateral sclerosis, Network, EEG, Cognition, Source localisation, Mismatch negativity, LS, Amyotrophic Lateral Sclerosis, fMRI, Functional magnetic resonance imaging, MMN, Mismatch negativity, EEG, Electroencephalography, qEEG, Quantitative EEG, MEG, Magnetoencephalography, PET, Positron emission tomography, IFG, Inferior frontal gyrus, MTG, Mid temporal gyrus, STG, Superior temporal gyrus, CWIT, Colour-word interference test, IQR, Interquartile range, AEP, Auditory evoked potential, LCMV, Linearly constrained minimum variance, eLORETA, Exact low-resolution brain electromagnetic tomography, AAL, Automated Anatomical Labelling, AUROC, Area under receiver operating characteristic curve, DLPFC, Dorsolateral prefrontal cortex

## Abstract

**Objective:**

To localise and characterise changes in cognitive networks in Amyotrophic Lateral Sclerosis (ALS) using source analysis of mismatch negativity (MMN) waveforms.

**Rationale:**

The MMN waveform has an increased average delay in ALS. MMN has been attributed to change detection and involuntary attention switching. This therefore indicates pathological impairment of the neural network components which generate these functions. Source localisation can mitigate the poor spatial resolution of sensor-level EEG analysis by associating the sensor-level signals to the contributing brain sources. The functional activity in each generating source can therefore be individually measured and investigated as a quantitative biomarker of impairment in ALS or its sub-phenotypes.

**Methods:**

MMN responses from 128-channel electroencephalography (EEG) recordings in 58 ALS patients and 39 healthy controls were localised to source by three separate localisation methods, including beamforming, dipole fitting and exact low resolution brain electromagnetic tomography.

**Results:**

Compared with controls, ALS patients showed significant increase in power of the left posterior parietal, central and dorsolateral prefrontal cortices (false discovery rate = 0.1). This change correlated with impaired cognitive flexibility (rho = 0.45, 0.45, 0.47, *p* = .042, .055, .031 respectively). ALS patients also exhibited a decrease in the power of dipoles representing activity in the inferior frontal (left: *p* = 5.16 × 10^−6^, right: *p* = 1.07 × 10^−5^) and left superior temporal gyri (*p* = 9.30 × 10^−6^). These patterns were detected across three source localisation methods. Decrease in right inferior frontal gyrus activity was a good discriminator of ALS patients from controls (AUROC = 0.77) and an excellent discriminator of *C9ORF72* expansion-positive patients from controls (AUROC = 0.95).

**Interpretation:**

Source localization of evoked potentials can reliably discriminate patterns of functional network impairment in ALS and ALS subgroups during involuntary attention switching. The discriminative ability of the detected cognitive changes in specific brain regions are comparable to those of functional magnetic resonance imaging (fMRI).

Source analysis of high-density EEG patterns has excellent potential to provide non-invasive, data-driven quantitative biomarkers of network disruption that could be harnessed as novel neurophysiology-based outcome measures in clinical trials.

## Highlights

• Bilateral IFG and left STG activity is reduced in ALS.
• Left posterior parietal, motor and DLPFC activity is increased in ALS.
• Greater frontoparietal power correlates to longer inhibition-switching time in ALS.
• IFG and STG activity has good discrimination ability between ALS and controls.
• IFG activity has excellent discrimination ability between C9orf72+ ALS and controls.

## 1 Introduction

Amyotrophic lateral sclerosis is a progressive neurodegenerative condition characterized by upper and lower motor neuron degeneration (Kiernan et al., 2011). Extra-motor behavioural and cognitive symptoms are common in ALS (Phukan et al., 2007), and imaging technologies have provided early evidence of broader network disruption (Bede et al., 2015, Bede et al., 2018).

Although structural imaging can reliably record changes in grey and white matter integrity (Schuster et al., 2016) and functional imaging detects resting and activated states of metabolic activity (Erdoğan et al., 2016), there remains an unmet need for real-time measurement of different patterns of network disruption.

### 1.1 EEG for assessing neural function

Electrophysiological measurement of network activity during cognitive performance allows for direct objective quantification of dysfunction (Katada et al., 2004) with excellent temporal resolution (Teplan, 2002). These measures, captured by EEG or MEG, are distinct from secondary blood flow or oxygen content measures upon which fMRI is based (Erdoğan et al., 2016). EEG measures the electrical dipoles produced by transmembrane ion flow in large numbers of simultaneously-active, aligned cortical neurons, while MEG measures the concurrently generated magnetic fields (da Silva, 2013). EEG/MEG measurements have traditionally been limited by noise from extracerebral (such as facial muscles, ocular, and cardiac) artefacts, in addition to poor spatial resolution (Reis et al., 2014). However, the use of improved recording instrumentation with up to 256 sensors, combined with digitized data processing (Dukic et al., 2017; Muthuraman et al., 2018; Nasseroleslami et al., 2017), has substantially improved the signal to noise ratio.

Due to volume conduction, EEG sensors capture electrical currents propagated from both adjacent and distant sources in the conductive human head medium. However, source localisation of EEG sensor recordings localises the activity underlying these signals with spatial resolution comparable to fMRI (Moeller et al., 2013) and source localised MEG (Muthuraman et al., 2014). Furthermore, as EEG does not require expensive superconductive systems needed for MEG (Wendel et al., 2009), it is more cost effective and therefore more suited to day-to-day clinical application.

As EEG/MEG directly measure the functional neuronal activity at a network level, they can capture cognitive network dysfunction in the absence of cognitive symptoms (Döring et al., 2016), and therefore may provide greater sensitivity to cognitive pathology than psychological (behavioural) task parameters. Correlating these measures with specific domains of cognitive impairment could provide quantifiable cognitive biomarkers to improve neurodegenerative disease diagnostics, and additional outcome measures for clinical trials.

### 1.2 MMN an index of cognitive decline

MMN is a measure typically elicited and recorded during an auditory oddball task, wherein the participant receives series of auditory stimuli (tones). These tones are of one pitch, except for a fraction of cases (e.g. 10% in this study) which are of higher “deviant” frequency (pitch). The MMN is a negative waveform, found by the difference between the auditory evoked potentials generated by these deviant and standard tones at 100-300 ms post-stimulus (Iyer et al., 2017; Näätänen et al., 2007; Naatanen, 1995).

Multiple hypotheses have been proposed regarding the cortical function(s) measured by MMN, including both sensory and cognitive components of auditory processing. MMN was first described by Näätänen et al. in 1978, who hypothesised that the waveform resulted from comparison of a deviant input to a sensory memory template. It was also suggested that MMN might represent recognition of target criteria fulfilment (Näätänen et al., 1978), however such a “relevance effect” was considered unlikely as attention to the stimulus did not affect the waveform (Näätänen, 1995). This was subsequently supported by multiple studies demonstrating MMN in the absence of attention (Winkler et al., 1996), including in sleeping infants (Ruusuvirta et al., 2009) or those in a vegetative state (Wijnen et al., 2007). The MMN was therefore proposed to reflect an automatic detection of sensory change and modification of the physiological model of the environment to incorporate this new stimulus, referred to as the *model adjustment hypothesis* (Winkler et al., 1996).

An additional automatic attention-switching process related to the frontal generators was then proposed to occur on the basis that right frontal sources were activated irrespective of the ear detecting the stimulus change (Giard et al., 1990). This is believed to reflect the call to switch attention to changes in the unattended environment (Winkler et al., 1996), the occurrence of which is supported by autonomic responses such as heart rate and skin conductance changes following MMN (Lyytinen et al., 1992) as well as many other studies (Escera et al., 2001, Escera et al., 2003; Schröger, 1996).

An alternative *adaptation hypothesis,* first proposed by May et al. in 1999 (May et al., 1999; May and Tiitinen, 2001, May and Tiitinen, 2004), proposes that the MMN response results from cortical adaptation to monotonous stimuli, with MMN reflecting the difference between N1 to a novel sound and a lower amplitude, higher latency N1 generated by repetitive standard tones. This hypothesis was supported by later studies, such as those of Jääskeläinen et al. (Jääskeläinen et al., 2004) and Ulanovsky et al., (Ulanovsky et al., 2003) (for review see (May and Tiitinen, 2010)). However, an exclusively auditory hypotheses cannot account for the established prefrontal activation during MMN. Indeed, source localisation of PET, EEG, fMRI and MEG-derived MMN has reliably highlighted both the superior temporal and inferior frontal gyri as important sources of this signal (Rinne et al., 2000; Opitz et al., 2002; Yago et al., 2001; Müller et al., 2002), demonstrating that volume conduction alone does not account for frontal MMN. Furthermore, those with lesions of the dorsolateral prefrontal cortex have also been found to have reduced MMN amplitudes (Alho et al., 1994).

Source localisation across the MMN timeframe has additionally revealed two subcomponents, an early, sensory component that is maximal in the late N1 range (105-125 ms post-stimulus) generated by temporal sources and a later, cognitive component (170-200 ms post-stimulus), generated by frontal and temporal sources (Giard et al., 1990; Rinne et al., 2000; Maess et al., 2007). These temporal sources are attributed to sensory memory and change detection while the later active, frontal sources are attributed to involuntary attention switching in response to change (Giard et al., 1990; Rinne et al., 2000; Alho, 1995; Näätänen and Michie, 1979). As this early component overlaps with the N1 range, temporal activity may also represent sensory detection (May and Tiitinen, 2010).

Hence, source-localised MMN affords the benefit of separately interrogating each of these functions and the neural network which generate them, both in healthy individuals and those with neurological diseases. This is supported by several previous studies in different neurological and neuropsychiatric diseases, where MMN has been used as an index of abnormal auditory perception, involuntary attention switching, pathological brain excitability and cognitive and functional decline (see (Näätänen et al., 2012, Näätänen et al., 2014; Schall, 2016; Todd et al., 2013; Kujala and Leminen, 2017) for reviews).

### 1.3 Identifying the sources of MMN change in ALS

Using qEEG to measure MMN, we recently have shown a functional change in the underlying networks in ALS, with MMN being significant in healthy controls from 105 to 271 ms post-stimulus and having an increased average delay within the 100-300 ms post-stimulus window in ALS (Iyer et al., 2017). Due to the limited spatial resolution of sensor space studies, however, the specific sources contributing to MMN change and the nature of their dysfunction in ALS remains unclear. We therefore were unable to specify which network components indexed by MMN are affected by ALS pathology.

In this study we have used high-density qEEG in combination with each of three source localisation methods to determine and cross-validate the locations of MMN generators, and to measure differences in their activity between ALS patients and healthy controls. Here we show how the dysfunction of each source of MMN is affected by ALS, characterized by both under-active and over-active sources contributing to the abnormal response.

## 2 Methods

### 2.1 Ethical approval

Ethical approval was obtained from the ethics committee of Beaumont Hospital (REC reference: (Nasseroleslami et al., 2017)/102) and the St. James's Hospital (REC reference: 2017–02). All participants provided written informed consent before participation. All work was performed in accordance with the Declaration of Helsinki.

### 2.2 Participants

#### 2.2.1 Recruitment

Patient recruitment was undertaken from the National ALS specialty clinic in Beaumont Hospital. Healthy controls included neurologically-normal spouses of ALS patients and neurologically-normal, age- and sex-matched individuals recruited from an existing cohort of population-based controls.

#### 2.2.2 Inclusion criteria

Patients were over 18 years of age and diagnosed within the previous 18 months with Possible, Probable or Definite ALS in accordance with the El Escorial Revised Diagnostic Criteria.

#### 2.2.3 Exclusion criteria

Patients with Transient Ischemic Attack, Multiple Sclerosis, stroke, seizure disorders, brain tumours, structural brain diseases and other comorbidities were excluded.

#### 2.2.4 Demographics of patients and controls

A total of 95 ALS patients and 43 controls underwent recording. 58 ALS patients (f/m: 20/38; age: 59.2 years, range: 29–81 years) and 39 healthy controls (f/m: 28/11; age: 58.9 years, range: 36–78 years) were included in final analyses. Data with poor recording quality (determined by the lack of auditory evoked potentials), were excluded. Eight controls and 44 patients were also included in our previous sensor-space analysis (Iyer et al., 2017).

#### 2.2.5 Medical profile

Within the ALS group, 44 patients had spinal onset, 12 bulbar, and 2 thoracic onset. All patients were tested for the hexanucleotide repeat expansion in *C9ORF72*, of whom 7 were positive (*C9ORF72*+). Twelve patients had a known family history of at least one first or second degree relative with ALS, 3 of whom carried the *C9ORF72* repeat expansion. One additional patient had a known family history of at least one first or second degree relative with frontotemporal dementia (Byrne et al., 2012a). A contemporaneous ALSFRS-R score was available in 51 patients. Mean ALSFRS-R was 37.8 with an IQR of 33.5–42. Mean disease duration was 1.83 years (IQR: 0.89–2.09) from estimated symptom onset.

### 2.3 Experimental paradigm

EEG was recorded across 128-channels in 3 consecutive, 8 min sessions, during which an auditory frequency-mismatch oddball paradigm was delivered as described in our previously reported methods (Iyer et al., 2017). In total, 1350 standard trials and 150 deviant trials were presented.

### 2.4 Data acquisition

EEG recordings were conducted in the Clinical Research Facility at St. James's Hospital, Dublin using a BioSemi® ActiveTwo system (BioSemi B.V., Amsterdam, The Netherlands) within a Faraday cage. Subjects were measured with an appropriately-sized EEG cap. Data were online filtered to a bandwidth of 0–134 Hz and digitized at 512 Hz. Common average referencing was used. 27 patients also undertook the Colour-Word Interference Test from the Delis-Kaplan Executive Function System (Delis et al., 2001), which is a test of attention shift, inhibitory control, error monitoring and cognitive flexibility.

### 2.5 Data analysis

Data were preprocessed as described in our previous sensor space analysis (Iyer et al., 2017) using custom MATLAB (version R2014a and R2016a, Mathworks Inc., Natick, MA, USA) scripts and the FieldTrip Toolbox (Oostenveld et al., 2011). Mean number of included artefact-free standard/deviant trials was 1267/144 for patients and 1223/146 for controls. For source analyses the number of standard trials was matched to that of deviant trials by random selection.

### 2.6 EEG signal processing

The mean standard and deviant auditory evoked potentials were calculated for each participant from 100 ms before the stimulus to 500 ms post-stimulus as previously reported (Iyer et al., 2017). MMN waveforms were calculated for each electrode in each individual as the difference between mean deviant and standard AEPs. Channels with continuously noisy data were excluded (mean excluded channels ± standard deviation in controls: 1.59 ± 1.65, patients: 1.52 ± 1.55) and data from these channels were modelled by spline interpolation of neighbouring channels.

### 2.7 Source localisation and analysis

Source localisation was implemented using custom MATLAB (version R2016a) scripts and the FieldTrip Toolbox for linearly constrained minimum variance (Van Veen et al., 1997) beamforming and dipole fitting, as well as LORETA-KEY software (version 20170220, The KEY Institute for Brain-Mind Research, Zurich, Switzerland) for exact low-resolution electrotomography (Pascual-Marqui et al., 2011). Three different source localisation methods were used to circumvent the limitations imposed by different mathematical assumptions for finding a unique solution to the ‘inverse problem’ by each single method (Darvas et al., 2004) (Table 1). Head models incorporating individual geometries for the brain, skull and scalp tissues were constructed for 41 patients. Boundary-element head models (Fuchs et al., 2002) were generated using T1 images from contemporaneous MRI (3-Tesla Philips Achieva scanner, Best, The Netherlands), acquired at the Centre of Advanced Medical Imaging, St. James' Hospital (Schuster et al., 2016). For other subjects with no personal MRI, the ICBM152 head model (Fonov et al., 2011) was used, as template-based and individualised boundary-element head models are found to provide comparable localisation accuracy (Fuchs et al., 2002; Douw et al., 2018).

**Table 1:** Limitations and advantages of different source localisation methods.

Method	Dipole fitting	LCMV	eLORETA
Spatial resolution	Excellent	Good	Low
Temporally correlated source detection	No limitation	Limited	No limitation
Prior knowledge required	Yes	No	No
Full brain map estimate	No	Yes	Grey-matter

#### 2.7.1 Linearly constrained minimum variance (LCMV)

LCMV is a beamforming source localisation method wherein the covariance of the signals recorded from the electrodes is used to generate a spatial filter formed by a linear combination of electrode weights, for each grid point in the brain. The identified solution is that which affords minimum experimental variance of data when projected to the source, thus minimising the amount of activity from other sources (Van Veen et al., 1997). LCMV was used to calculate brain maps of mean power for the average AEP 100-300 ms after standard and deviant cues, based on a common spatial filter. A time window of 100-300 ms was utilised to ensure accurate calculation of the covariance matrix from which the spatial filter is calculated and avoid high functional correlation between the sources which would hinder localisation of such distinct sources. Covariance matrices were also calculated for individual trials to minimise such correlations. Regularisation of the covariance matrix was implemented at 5% of the average variance of EEG electrodes to account for reduced dimensionality caused by independent component analysis during preprocessing. Sources within the brain volume were modelled by a grid with 10 mm resolution. The leadfield matrix was normalised to avoid potential norm artefacts. Sources of MMN activity were identified by the locations of the maximal logarithm of the power ratio between deviant and standard maps.

#### 2.7.2 Exact low resolution brain electromagnetic tomography (eLORETA)

ELORETA (Pascual-Marqui et al., 2011) identifies a unique source power map based on the implicit assumption that neighbouring dipoles have similar activity (low spatial resolution). This is achieved by identifying the solution with the least activity norm, subject to minimising the Laplacian (spatial gradient or derivative) of the sources. This assumption yields solutions with a relatively low spatial resolution. ELORETA was also used to calculate mean source power maps of the average auditory evoked potential 100-300 ms after standard and deviant cues to match the data input to LCMV. LORETA-KEY software models sources at 5 mm resolution within the brain volume of a boundary-element headmodel based on the Colin27 average brain (Holmes et al., 1998), excluding sources located within white matter. For statistical comparison, grid resolution was reduced to 10 mm to avoid the loss of discriminatory power that may result from correction of over 6000 comparisons. Regularisation was implemented for a signal to noise ratio of 10. Sources of MMN activity were identified as described for LCMV.

#### 2.7.3 Dipole fitting

Dipole fitting can be used to generate least-square error models of the contributions of electrical dipoles to an EEG topographic distribution, given a-priori estimation of the number and location of contributing dipoles (Scherg and Berg, 1991). Residual variance (the variance in the data not explained by the model) is used as a goodness-of-fit measure. Previous studies (Jemel et al., 2002; Oknina et al., 2005; Oades et al., 2006) have repeatedly identified MMN sources in the inferior frontal gyri and superior temporal gyri. As non-linear optimisation of the dipole location repetitively produced fits at local rather than global residual variance minima, four fixed dipoles were modelled at the centroid coordinates of the bilateral superior temporal gyri and pars triangularis of the inferior frontal gyri, as determined from an AAL atlas (Tzourio-Mazoyer et al., 2002). Models were estimated based on the average MMN response (mean{deviant response}-mean{standard response}) for 40 ms surrounding the global field power peak between 105 and 271 ms post-stimulus, the period for which we previously found MMN to be significant (Iyer et al., 2017). Subsequently, mean power for each dipole was calculated. The rationale for using this shorter time frame was based upon findings that these four sources better accounted for the data in this window (i.e. had smaller residual variance) than the longer time window of data 100-300 ms post-stimulus, as used for LCMV and eLORETA. A model generated using the longer 200 ms time window provided the same results as the model reported here.

### 2.8 Statistics

#### 2.8.1 LCMV and eLORETA

A 10 mm grid in the brain volume yields 733 sources excluding white matter (as modelled by eLORETA) and 1726 sources including white matter (as modelled by LCMV). To analyse these high-dimensional data, 10% False Discovery Rate (Benjamini, 2010) was used as a frequentist methods for preliminary screening. Subsequently, Empirical Bayesian Inference (EBI) (Efron, 2009) was used to find Bayesian posterior probabilities, as well as achieved statistical power and AUROC. AUROC is a measure of how well the test separates patient and control groups (Hajian-Tilaki, 2013) which ranges from 0 to 1, where if the null hypothesis of no separation is true, AUROC equals 0.5. Therefore, the further the value of AUROC from 0.5, the greater the separation.

#### 2.8.2 Dipole fitting

Dipole power for each of the four modelled dipoles in the complete ALS group as well as *C9ORF72*+, *C9ORF72*−, bulbar-onset and spinal-onset subgroups were compared by Mann-Whitney *U* test. Bonferroni correction for multiple comparisons established a significance threshold of *p* < .0025. AUROC and statistics were also calculated for each dipole by empirical bootstrapping-based inference (Nasseroleslami, 2018).

#### 2.8.3 Neuropsychology correlation

Spearman's rank partial correlation (which is inherently robust to outliers) was used to individually compare changes in EEG source power to CWIT performance (colour naming, word reading, inhibition and inhibition switching times in seconds) while correcting for speech impairment (ALS-FRS speech score on the day of testing) and age. CWIT was investigated on the basis of a previously identified correlation between sensor-level MMN average delay and performance in this task (Iyer et al., 2017). Correlations were performed for power in each fitted dipoles and for the mean power in the left superior and medial frontal gyri (combined), primary motor cortex and posterior parietal cortex, according to the AAL atlas (Tzourio-Mazoyer et al., 2002). Multiple comparison correction was by Bonferroni correction. Beaumont Behavioural Inventory (Elamin et al., 2017) and Edinburgh Cognitive Assessment Score (Pinto-Grau et al., 2017) data were also available, however the main scores of these measures showed no significant correlation to source activity and were, therefore, not investigated further.

## 3 Results

### 3.1 Dipole fitting

**Fig. 1:**
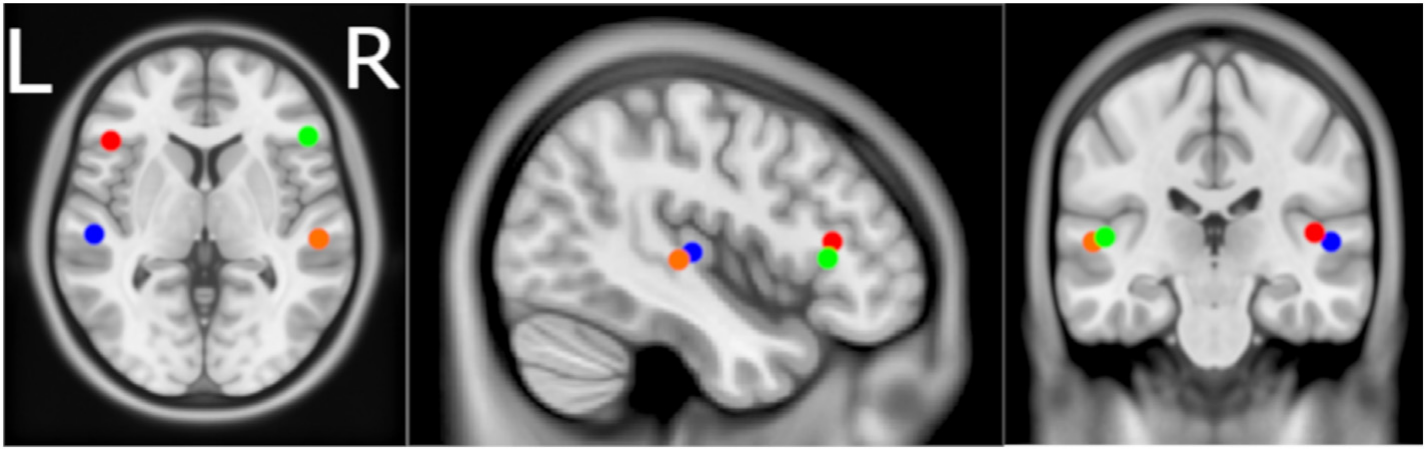
Location of dipoles modelled by dipole fitting. Centroids of the left (blue) and right (orange) superior temporal gyri and left (red) and right (green) inferior frontal pars triangularis were used to seed dipoles for dipole fitting. Axial MRI view is from above (L-Left, R-Right).

**Fig. 2:**
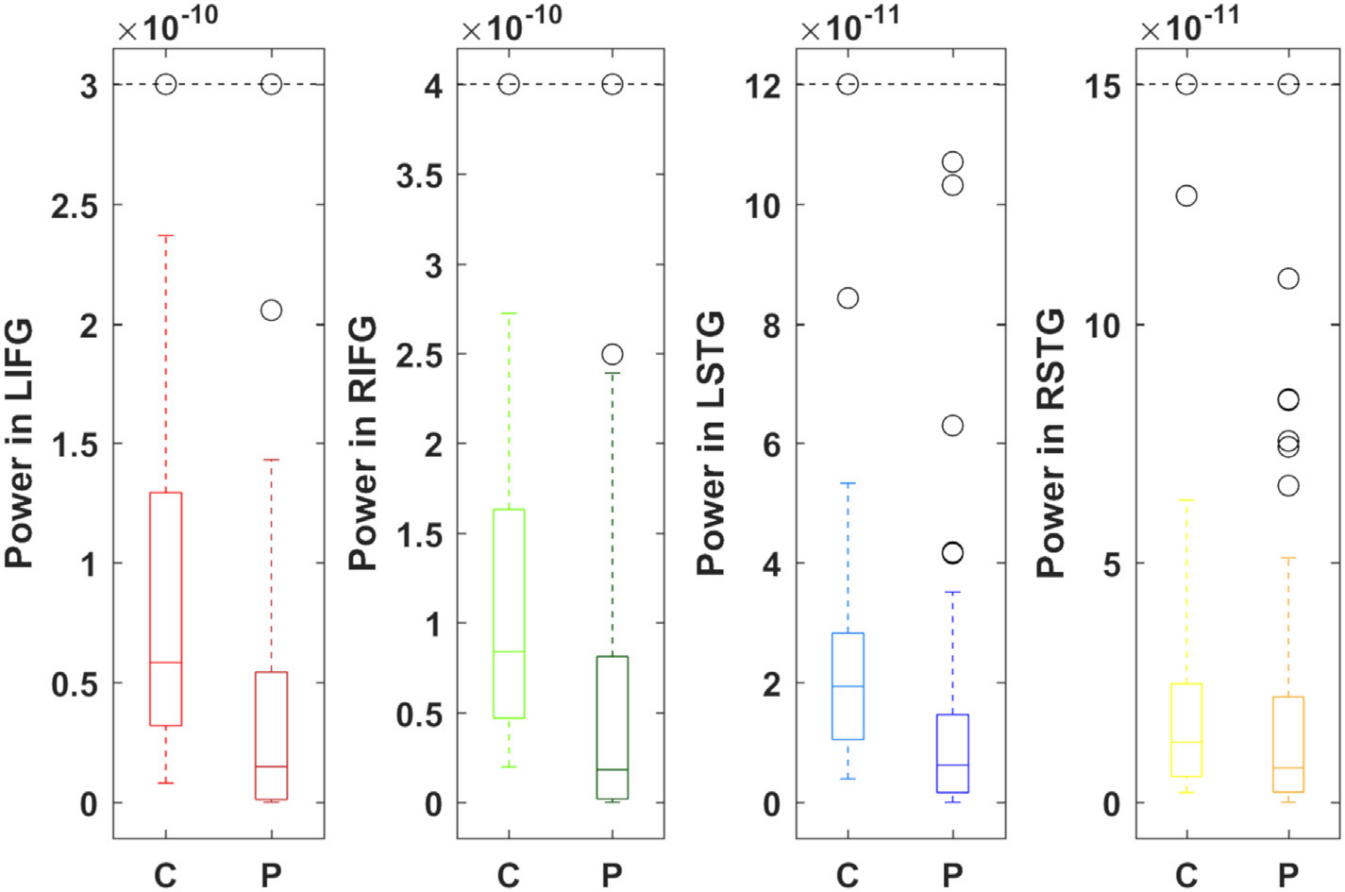
ALS patients show decreased power in both inferior frontal gyri and the left superior temporal gyrus. Boxes illustrate the interquartile range with whiskers illustrating the maximum and minimum power (A-m) within twice the interquartile range for ALS patients (P) and controls (C), determined by dipole fitting. Outliers are illustrated in black. Dashed line caps up to two outliers beyond this value. L – Left, R – Right, IFG – Inferior frontal gyrus, STG – Superior Temporal Gyrus.

Locations of dipole fits are illustrated in Fig. 1. Control and patient groups showed similar goodness of fit (median (IQR): patients: 23.32% (15.24–30.2%), controls: 24.39% (15.55–35.49%)). *P*-values obtained by Mann-Whitney *U* test comparison of dipole power between ALS patients and healthy controls are summarised in Table 2. Power was significantly lower in the IFG bilaterally as well as the left STG. AUROC demonstrated that power in each of these three dipoles has good group discrimination ability (Table 2, Fig. 2). No differences were found between male and female patients for any dipole (*p* = .27–.75, AUROC = 0.42–0.58). The discrepancy from complete fit indicated the presence of additional sources, which were subsequently aggregated by eLORETA and LCMV.

**Table 2:** Summary of P-values and AUROCs for each source modelled by dipole fitting in ALS patients and subgroups compared to controls. All subgroups show decreased power in inferior frontal and left temporal dipoles compared to controls. Inferior frontal activity has excellent discrimination ability between *C9ORF72*+ patients and controls and good discriminating ability in other groups. P-values were obtained by Mann-Whitney U test. AUROC given in parentheses. Bold indicates statistical significance (*p* < .0025).

Dipole Location	All	C9orf72+	C9orf72-	Bulbar-onset	Spinal-onset
Left IFG	**5.16 × 10**^**−6**^	**6.87 × 10**^**−4**^	**1.98 × 10**^**−5**^	**1.22 × 10**^**−3**^	**1.22 × 10**^**−3**^
	(0.7741)	(0.9084)	(0.7637)	(0.802)	(0.769)
Right IFG	**1.07 × 10**^**−5**^	**2.15 × 10**^**−4**^	**9.29 × 10**^**−5**^	**2.37 × 10**^**−5**^	**1.74 × 10**^**−4**^
	(0.7648)	(0.9451)	(0.7416)	(0.895)	(0.74)
Left STG	**9.30 × 10**^**−6**^	0.016	**2.30 × 10**^**−6**^	2.64 × 10^−3^	**2.40 × 10**^**−4**^
	(0.7666)	(0.7912)	(0.761)	(0.795)	(0.738)
Right STG	0.081	0.39	0.118	0.035	0.23
	(0.6052)	(0.6044)	(0.5968)	(0.698)	(0.576)

### 3.2 eLORETA

**Fig. 3:**
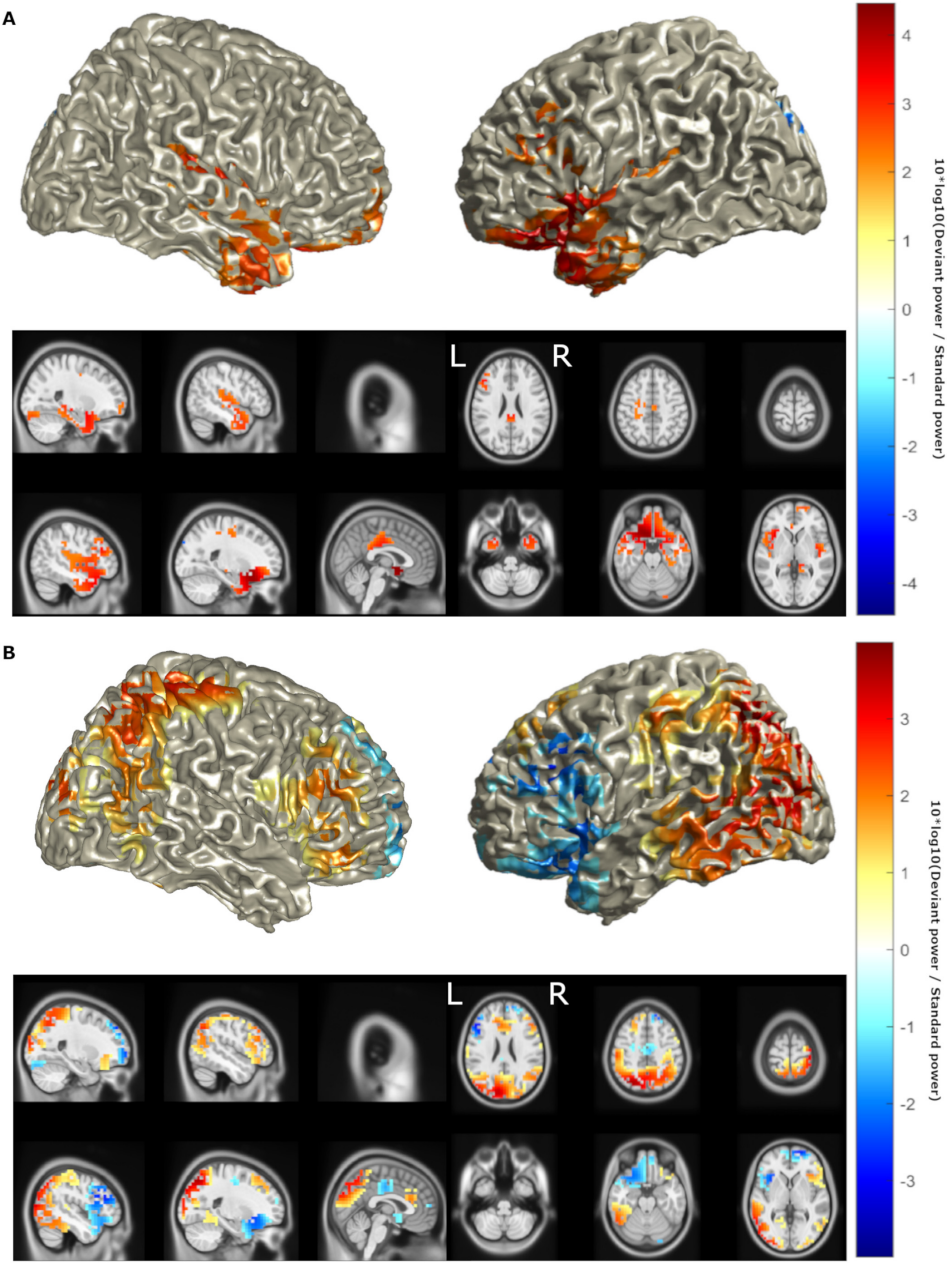
ELORETA identified a pattern of decreased activity in the left superior temporal and inferior frontal sources, and an increase in activity in posterior areas. Location of MMN sources with (a) top 50% of power (10*log_10_(Deviant power / Standard power)) in healthy controls and (b) power differences >25% of maximum between ALS patients and healthy controls as determined by eLORETA. Red denotes increase in power, blue denotes decrease in power. Axial MRI views are from above (L-Left, R-Right).

ELORETA identified maximal intensity of neural activity during MMN in the left IFG and bilateral STG and MTG in controls (Fig. 3a), confirming the localisation of major sources to those previously established, with the exception of the right IFG (Jemel et al., 2002; Oknina et al., 2005; Oades et al., 2006). ALS patients showed a pattern of reduced activity in these sources, consistent with the results of dipole fitting, as well as an increase in activity in posterior sources (Fig. 3b). While the eLORETA estimated the general distribution pattern of activity, the method's low spatial resolution prevented the effects reaching statistical significance.

### 3.3 LCMV

**Fig. 4:**
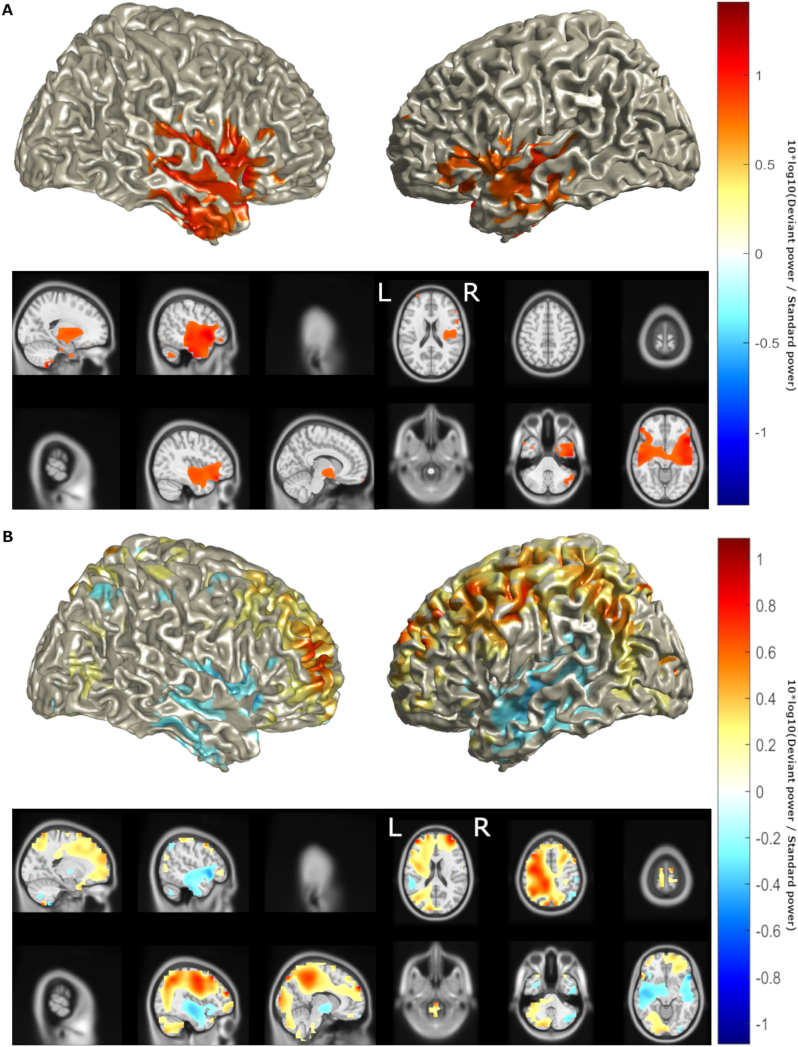
LCMV identified a pattern of decreased activity in bilateral superior temporal and inferior frontal sources, and an increase in activity in the left hemisphere. Location of MMN sources with (a) top 25% of power (10*log_10_(Deviant power / Standard power)) in healthy controls and (b) power differences >25% of maximum between ALS patients and healthy controls as determined by LCMV beamforming. Red denotes increase in power, blue denotes decrease in power. Axial MRI views are from above (L-Left, R-Right).

**Fig. 5:**
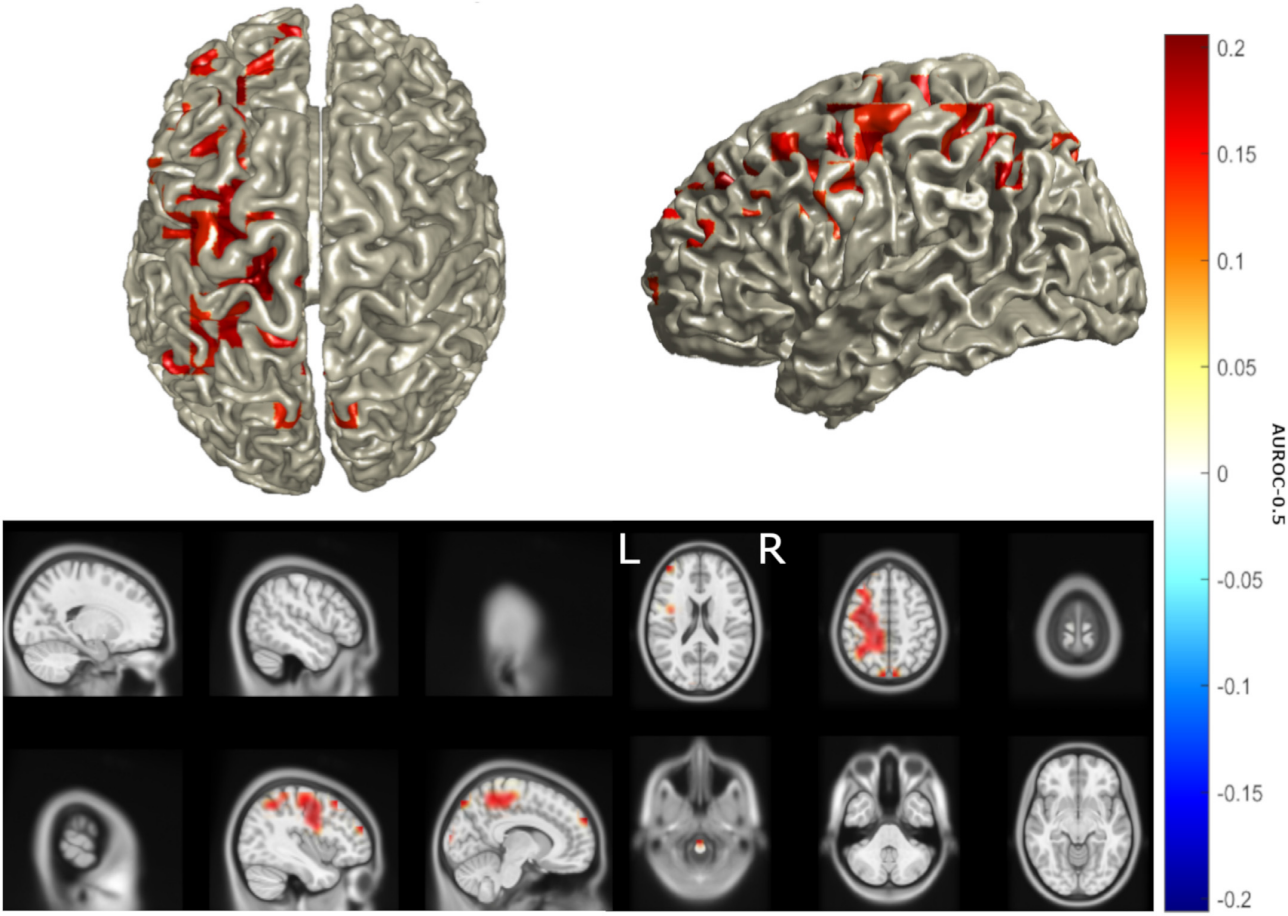
Increased activity in the left posterior parietal, central and dorsolateral prefrontal cortex in ALS is statistically significant. Statistically significant (false discovery rate = 10%) differences in power between ALS patients and healthy controls as determined by LCMV. Heat map values are AUROC-0.5. Red denotes AUROC>0.5, blue denotes decrease in AUROC<0.5. Axial MRI views are from above (L-Left, R-Right).

**Fig. 6:**
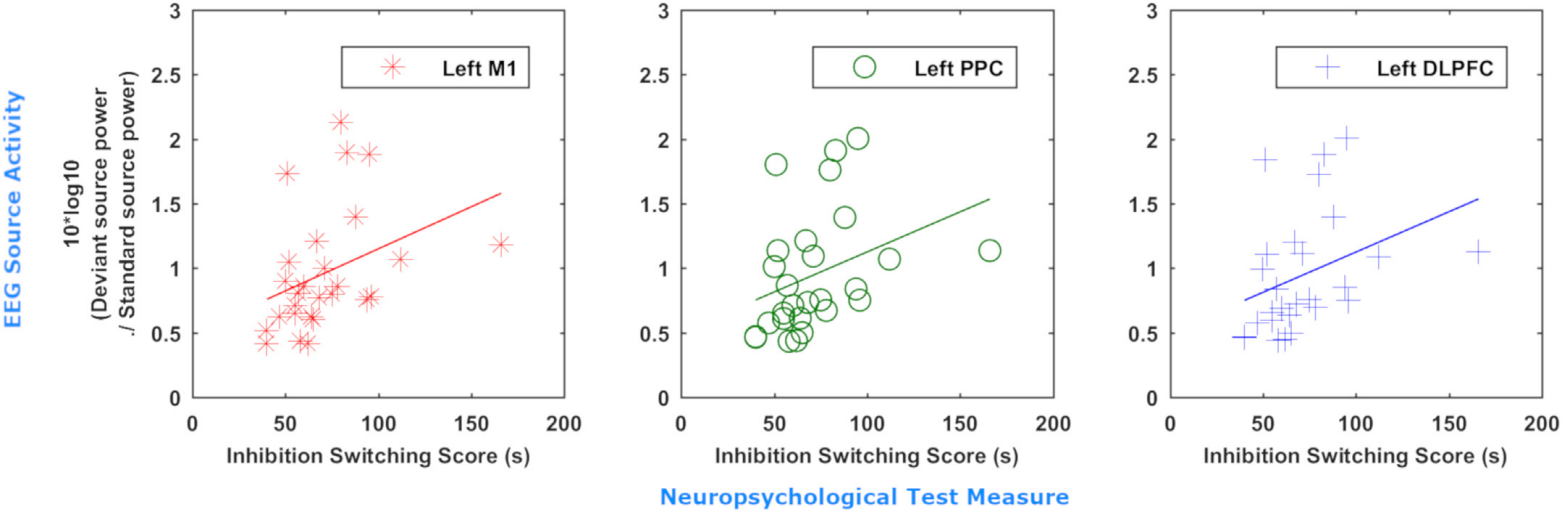
Increased activity in the posterior parietal and dorsolateral prefrontal cortex correlates to poorer performance in cognitive switching tasks. Correlation of inhibition/switching score (in seconds) for 27 patients with mean power in the left primary motor cortex (red), posterior parietal cortex (PPC, green), and middle and superior frontal gyri (M/SFG, blue) illustrated by scatterplot with line of best fit.

LCMV identified sources of MMN similar to the findings of eLORETA (Fig. 4a) but also identifying the right IFG as a source, as identified by previous studies (Jemel et al., 2002; Oknina et al., 2005; Oades et al., 2006). LCMV also detected a trend of reduced activity in these sources bilaterally, in keeping with the results of dipole fitting and eLORETA, as well as an increase in activity in the left parietal, central and dorsolateral prefrontal cortex (Fig. 4b). This increase reached statistical significance (Fig. 5, FDR = 10%, statistical power = 0.58). Based on interpolation with an AAL atlas, sources with significantly increased activity included the superior parietal lobe and precuneus, left motor structures including the primary motor cortex, supplementary motor area and mid cingulum, as well as the mid frontal gyrus (Table 3). Positive correlations (Fig. 6) were found between CWIT inhibition-switching time (but not other CWIT scores) and mean power in the left primary motor cortex (ρ = 0.45, *p* = .055), the superior and middle frontal gyri combined (ρ = 0.47, *p* = .031) and the posterior parietal cortex (ρ = 0.45, *p* = .042), where greater inhibition-switching score indicates more impaired cognitive flexibility and verbal inhibition (Swanson, 2005). *P*-values below 0.05 in the prefrontal and parietal cortices did not survive multiple comparison correction, likely due to the low number of CWIT scores available. No significant differences were found between male and female patient sources (α_global_ = 0.92, β _global_ = 0.075) or mean power of the left posterior parietal, motor or inferior frontal cortices (*p* = .56–.89).

**Table 3:** Comparison of the head and source models, time windows and detected source activity changes for each source localisation method used. L – left, R – right, IFG – inferior frontal gyrus, STG – superior temporal gyrus. Arrows represent direction of change in power. *Statistically significant (*p* < .0025). BEM – Boundary element model.

Method	Head/source model	Time (ms)	L IFG	R IFG	L STG	R STG	Other significant source changes
LCMV	ICBM152/personal MRI BEM, 10 mm grid	100–300	↓	↓	↓	↓	↑* Left superior parietal lobe, precuneus, primary motor cortex, supplementary motor area, mid cingulum, mid frontal gyrus
eLORETA	Colin27 MRI BEM, 10 mm grid excl. white matter	100–300	↓	↑	↓	↓	None
Dipole fitting	ICBM152/personal MRI BEM, 4 dipoles	105–271 & 100–300	↓*	↓*	↓*	↓	N/A

### 3.4 Differences between ALS subgroups

*C9ORF72*+ patients were not distinguished from *C9ORF72*− patients by any localisation method, nor were bulbar-onset from spinal-onset patients. This was likely due to insufficient sample size. *C9ORF72*− and spinal subgroups individually showed similar patterns of significant difference to the full patient group across each localisation method. Bulbar and *C9ORF72*+ subgroups significantly differed from controls with respect to bilateral IFG dipole activity, and exhibited better discrimination ability (summarised in Table 2). The discrimination ability of this difference was excellent for *C9ORF72*+ patients (AUROC>0.9) with low AUROC variation (0.002 bilaterally). CWIT and speech score data were insufficient (*C9ORF72*+ *n* = 0, bulbar-onset *n* = 3) for correlation analyses.

## 4 Discussion

This study demonstrates that source localization of cognitive event-related potentials measured by EEG reliably distinguishes attentional network changes in ALS patients compared to controls, particularly in subgroups with higher prevalence of cognitive impairment (Byrne et al., 2012b; Schreiber et al., 2005). Furthermore, this study indicates for the first time a correlation between the activities of specific sources underlying cognitive event-related potentials and cognitive performance in a neurodegenerative disease. Compared with controls, ALS patients show decreased activity in both inferior frontal gyri and the left superior temporal gyrus and increased left posterior parietal and dorsolateral prefrontal activity. ALS patients also show significantly increased activity in the left motor cortex.

### 4.1 Imbalance of attention-regulating network activity during sensory processing in ALS

The superior temporal and inferior frontal gyri are well established sources of MMN activity (Maess et al., 2007; Jemel et al., 2002; MacLean et al., 2015). In this study, decreased activity in these regions was identified independently using each of the methods, however, dipole fitting allowed for a more temporally and spatially precise interrogation of these sources.

Repetitive TMS (Nixon et al., 2004) and nonword rhyming task studies (Burton, 2001) have demonstrated the role of the IFG in phonological working memory, where information about one stimulus is stored for later comparison to a second. The IFG is also known to be active when ignoring stimuli (Bunge et al., 2001) and is functionally connected to the default mode network (Beaty et al., 2014). This network is active when directed attention is not required and is deactivated by goal-directed activity, as defined by resting-state fMRI (Raichle et al., 2001). The activity of the default mode network is anti-correlated with that of the central executive network, where attention needs to be directed to a task (Nekovarova et al., 2014). Inferior frontal source activity during the MMN is therefore consistent with calling for a switch of attention to changes in the unattended environment (i.e. involuntary attention switching), to which prefrontal MMN sources have previously been attributed (Winkler et al., 1996; Giard et al., 1990).

The observed substantial reduction in IFG activity in ALS is correspondingly expected to parallel impairments in these cognitive functions. As posterior parietal and dorsolateral prefrontal cortices are nodes of the central executive network (Seeley et al., 2007), their abnormal activation in combination with IFG dysfunction during MMN in ALS may represent a loss of balance between the activity of these attention-regulating networks (Menon and Uddin, 2010) resulting in dysregulation of involuntary attention switching.

As participants were asked to ignore and not respond to stimuli in this study, attention regulation could not be behaviourally measured during MMN recording. This hypothesis is, however, supported by our preliminary findings of a positive correlation between increases in left posterior parietal and dorsolateral prefrontal activity during MMN, and the inhibition/switching score of the CWIT (and not other subscores of the CWIT). This indicates that abnormal increase in the activity of this network conveys cognitive inflexibility and disinhibition (Swanson, 2005). Such behavioural inflexibility and disinhibition is consistent with incorrect orientation to irrelevant stimuli and is expected in those with abnormal central executive network activation. Correspondingly, change in bilateral IFG activity was shown to be an excellent discriminator of *C9ORF72*+ and bulbar-onset ALS subgroups, which are more prone to cognitive impairment (Byrne et al., 2012b; Schreiber et al., 2005).

This imbalance hypothesis is also evidenced by data from previously reported functional connectivity studies in ALS. For example, resting-state MEG has identified increased functional connectivity between the left posterior cingulate and prefrontal cortices, as well as within and between posterior parietal cortices, in addition to increased overall parietal connectivity (e.g. node weight) (Proudfoot et al., 2018). Furthermore, resting state fMRI has demonstrated increased left precuneus, posterior parietal and mid cingulate cortex connectivity in addition to decreased inferior frontal connectivity (Agosta et al., 2013) in ALS. Accordingly, the frontoparietal hyperactivity and inferior frontal depression observed in our study may reflect a spread in pathological hyperactivity into cognitive networks, which in turn alters the balance in normal network activity. Activation of the central cortex in addition to cognitive network nodes during MMN in ALS may correspondingly represent abnormal activation of networks connecting motor and cognitive areas. This is consistent with previous physiological studies which have consistently identified hyperactivity in upper motor neurons in ALS (Vucic and Kiernan, 2006) and loss of inhibitory control (Grieve et al., 2015).

ALS-FRS-R total score showed no correlation to source activity - this is likely a reflection of the relatively low burden disease in the majority of patients, and the study being underpowered to explore the subscores of ALSFRS-R. However, previous studies have shown that functional connectivity is increased with ALS and correlates with disease severity (Sorrentino et al., 2018). A reduction in MMN in healthy individuals is also found to parallel increased connectivity and decreased inhibitory control between underlying sources, particularly in frontal nodes (Cooray et al., 2014). The recently demonstrated relationship between cognitive impairment and disease stage in ALS (Crockford et al., 2018) is therefore likely to reflect the spread of hyperactivity from motor to cognitive networks.

### 4.2 Potentially abnormal function of auditory network in ALS

Temporal source activity has been attributed predominantly to sensory memory and change detection in early MMN (Giard et al., 1990; Rinne et al., 2000; Alho, 1995; Näätänen and Michie, 1979); however, it has also been found to contribute to MMN's later attention switching component (Maess et al., 2007). Furthermore, as the difference wave early in the 100-300 ms studied may also capture changes in N1 (May and Tiitinen, 2010), temporal activity may include sensory detection.

As STG contains the primary auditory cortex (Howard et al., 2000) and has been shown to be active during attention control (Hopfinger et al., 2000), the decrease in left STG activity identified here in ALS may represent impairment in either auditory or cognitive networks. These findings, in addition to the greater number of (excluded) patients lacking clear AEPs compared to controls, suggest the additional presence of auditory network dysfunction in ALS. An additional investigation of AEPs generated during a solely auditory task is required to investigate this network further in ALS.

### 4.3 Harnessing the advantages of quantitative EEG

The detected changes in ALS reflect the additive benefits of physiological investigation to those of structural imaging. The discriminative ability of these changes, determined by the AUROC (up to 0.95 here) was comparable to, or better than, that achieved by fMRI (AUROC = 0.714) (Welsh et al., 2013) and sensor space qEEG (AUROC = 0.69) (Iyer et al., 2017). This methodology therefore has the potential to provide neurodegenerative disease markers prior to the onset of discernible structural degeneration, allowing for earlier and more sensitive monitoring of potential interventions.

### 4.4 Limitations

A sample size of 58 patients and limited availability of psychological and clinical test scores restricted exploration of the relationship between cognitive symptoms and source activity within subgroups of this heterogeneous condition. Further studies of larger sample size are therefore warranted to explore such relationships and ALS inter-subgroup differences with greater statistical power.

### 4.5 Conclusion

In conclusion, combining multiple localisation methods to determine the sources of ERPs provides high spatial resolution to complement qEEGs' excellent temporal resolution in the investigation of ALS-related network dysfunction. The use of this approach to localise activity during other cognitive, motor and sensory tasks allows for detailed interrogation of the location and nature of brain network disruption in neurodegenerative disorder**s**, with the potential to provide early, non-invasive and inexpensive biomarkers of neurodegenerations or their subtypes.
